# *Enchytraeus albidus* Microarray: Enrichment, Design, Annotation and Database (EnchyBASE)

**DOI:** 10.1371/journal.pone.0034266

**Published:** 2012-04-27

**Authors:** Sara C. Novais, Joel Arrais, Pedro Lopes, Tine Vandenbrouck, Wim De Coen, Dick Roelofs, Amadeu M. V. M. Soares, Mónica J. B. Amorim

**Affiliations:** 1 Department of Biology & CESAM, University of Aveiro, Aveiro, Portugal; 2 Department of Electronics, Telecommunications and Informatics (DETI), Institute of Electronics and Telematics Engineering of Aveiro (IEETA), University of Aveiro, Aveiro, Portugal; 3 University of Antwerp, Department of Biology - E.B.T., Groenenborgerlaan, Antwerp, Belgium; 4 VU University Amsterdam, Institute of Ecological Sciences, De Boelelaan, The Netherlands; University of North Carolina at Charlotte, United States of America

## Abstract

*Enchytraeus albidus* (Oligochaeta) is an ecologically relevant species used as standard test organisms for risk assessment. Effects of stressors in this species are commonly determined at the population level using reproduction and survival as endpoints. The assessment of transcriptomic responses can be very useful e.g. to understand underlying mechanisms of toxicity with gene expression fingerprinting. In the present paper the following is being addressed: 1) development of suppressive subtractive hybridization (SSH) libraries enriched for differentially expressed genes after metal and pesticide exposures; 2) sequencing and characterization of all generated cDNA inserts; 3) development of a publicly available genomic database on *E. albidus*. A total of 2100 Expressed Sequence Tags (ESTs) were isolated, sequenced and assembled into 1124 clusters (947 singletons and 177 contigs). From these sequences, 41% matched known proteins in GenBank (BLASTX, e-value≤10^-5^) and 37% had at least one Gene Ontology (GO) term assigned. In total, 5.5% of the sequences were assigned to a metabolic pathway, based on KEGG. With this new sequencing information, an Agilent custom oligonucleotide microarray was designed, representing a potential tool for transcriptomic studies. EnchyBASE (http://bioinformatics.ua.pt/enchybase/) was developed as a web freely available database containing genomic information on *E. albidus* and will be further extended in the near future for other enchytraeid species. The database so far includes all ESTs generated for *E. albidus* from three cDNA libraries. This information can be downloaded and applied in functional genomics and transcription studies.

## Introduction

Enchytraeids (Oligochaeta), members of the soil mesofauna, play a key role on the regulation of the composition and activity of soil communities; they improve the soil pore structure and are involved in the organic matter decomposition [1]. *Enchytraeus albidus* is present in a wide range of soils and conditions worldwide. *E. albidus* have been increasingly used as indicators of soil health since the standardization of the ecotoxicological tests, where survival, reproduction and, more recently, bioaccumulation effects are measured [2]–[4]. There is ample literature on chemical and natural stress on enchytraeids at these levels e.g. with heavy metals [5], [6], organic substances [7], [8], chemical mixtures [9] and different soil properties [10]–[12]. Such information is of extreme importance as they provide the tools for risk assessors, and policy makers at a later stage. However, the current ecotoxicology tests are time consuming (e.g. 6 weeks for reproduction) and there is little mechanistic understanding of the impact caused by such stressors. Complementing existing knowledge with the molecular profiling and genomic studies can help considerably to elucidate modes of action, molecular pathways of response or general biological processes affected by stressors. Furthermore, it has been shown by several authors that responses at gene level can be observed in several invertebrates within short time intervals such as 1 or 2 days [13]–[17], presenting a clear advantage in comparison to the more time-consuming population studies.

Promising developments have taken place in this area in soil invertebrates with the establishment of Expressed Sequence Tag (EST) databases and microarrays for a few species of earthworms: *Lumbricus rubellus* [18] and *Eisenia fetida* [19] and the springtail *Folsomia candida* [20]. The generation of ESTs is of particular interest when studying non-genomic model organisms, which is the case of the referred invertebrate species and also *E. albidus*. This is an efficient way to retrieve sequence information on the protein coding part of the genome [20], although not comparable to present next generation sequencing techniques.

Regarding *E. albidus*, Amorim and co-authors started the EST sequencing project with a normalized cDNA library [16]. A cDNA microarray was developed based on this normalized library and has been used to study the effects of phenmedipham, copper, different soil properties or exposure duration [16], [21]–[23]. The existing cDNA library was improved using suppression subtractive hybridization-PCR (SSH-PCR), a technique that combines high subtraction efficiency with a normalization step to generate differentially expressed sequences equally represented in the library [24], [25].

In the present paper the following main points were addressed: 1) development of two SSH libraries enriched with genes differentially expressed after exposure to metals and pesticides at different concentrations and exposure times; SSH-metals was developed by exposure to cadmium, zinc, copper and nickel; SSH-pesticides was developed by exposure to dimethoate, atrazine, carbendazim and lindane; 2) sequencing and characterization of all generated cDNA inserts; 3) development of a publicly available genomic database on *E. albidus* including the ESTs, allowing the users to search e.g. for sequence similarity (BLAST), gene ontology terms and for sequence information on the differentially expressed genes at the different conditions. After assembling all the information, the existing microarray was enriched and developed into a denser populated Agilent custom oligonucleotide microarray. The present can be used for studies envisaging mechanistic understanding of stress and soil quality assessment.

## Development and Analysis

### cDNA Libraries Construction

**Figure 1:**
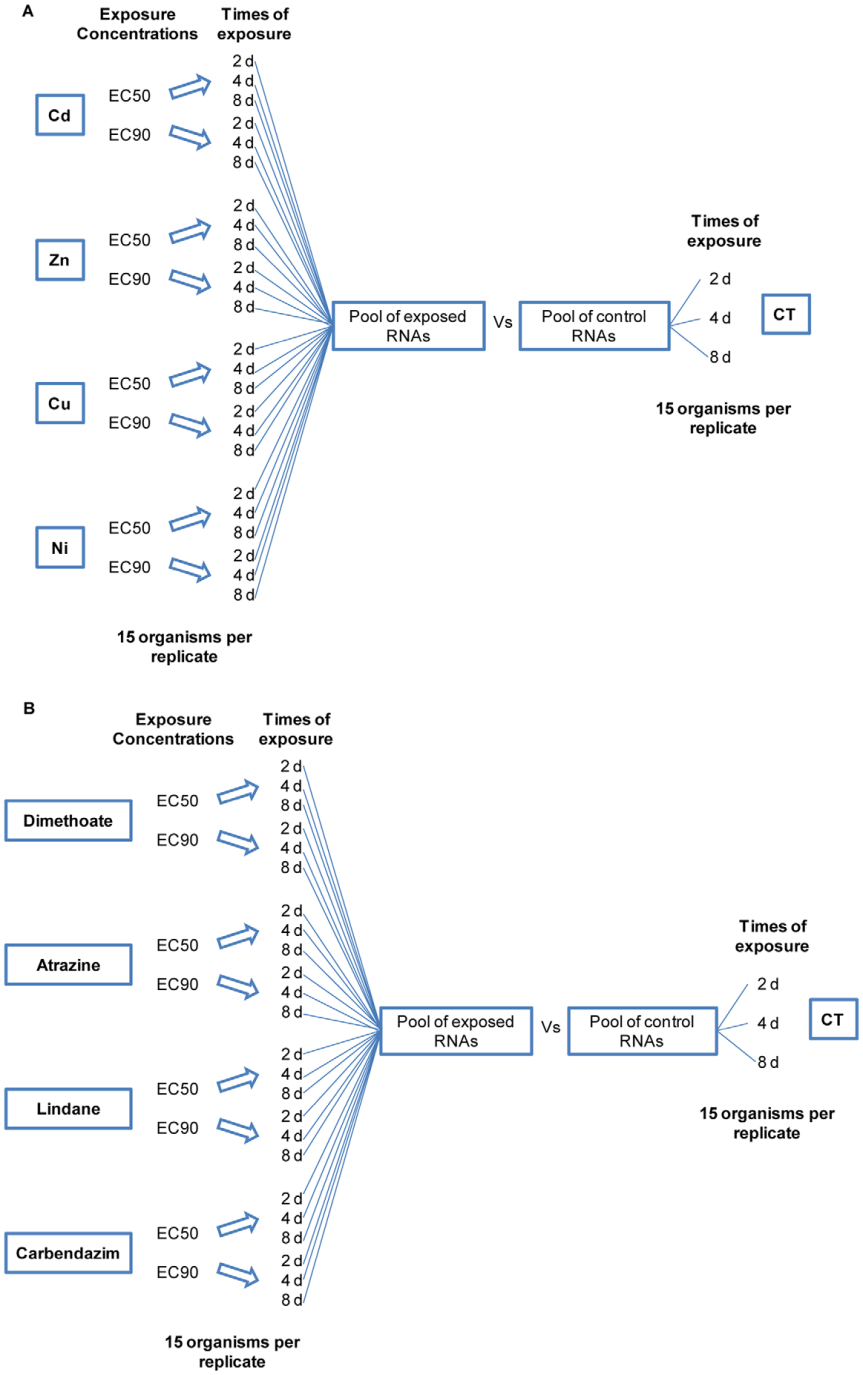
Exposure experimental setup. Schematic representation of the exposures performed and pools of RNA used for the SSH library development: A) Library enriched for genes differentially expressed after metal exposures; B) Library enriched for genes differentially expressed after pesticide exposures. CT = Control; EC50 = Concentration that induces 50% reduction in the number of juveniles (50% effect concentration on reproduction); EC90 = Concentration that induces 90% reduction in the number of juveniles (90% effect concentration on reproduction).

SSH procedure was applied for the development of two cDNA libraries (SSH-metals, SSH-pesticides) [24], [25]. A schematic representation of the exposures and RNA pools made for both SSH enriched cDNA libraries is shown in Figure 1.

For the library enriched with differentially expressed genes after metal exposures, 15 adult organisms with well developed clitellum were exposed, in each replicate, to 25 g of LUFA 2.2 standard natural soil [26], moist to 50% of the water holding capacity according to the standard guidelines [2], [3]. Soil was spiked with 4 different metal salts individually: cadmium chloride, zinc chloride, copper chloride and nickel chloride. Enchytraeids were exposed to each metal in two different concentrations in the range of the effective concentrations for 50% (EC_50_) and 90% (EC_90_) reduction in reproduction (known based on previous results [5], [27], [28]) and three time points (2, 4 and 8 days). These concentrations were selected, on the one hand to be able to relate gene effects with known effects at higher levels of biological organization and on the other hand to increase the likelihood of finding effects in gene expression, than would be expected with very low concentrations. Three replicates per condition were used. The total RNA from the organisms in each replicate was extracted using the Trizol extraction method (Invitrogen, Belgium). RNA concentration and purity was determined by spectrophotometry (NanoDrop 1000, Thermo Fisher Scientific) and quality was checked by denaturing formaldehyde agarose gel.

A pool containing RNA from all the exposure conditions was made using 1 replicate of each condition. A second pool containing RNA from control organisms (organisms exposed to clean LUFA 2.2 soil) was similarly prepared. For the library enriched for differentially expressed genes after pesticide exposures, enchytraeids were exposed to dimethoate, atrazine, lindane and carbendazim. Exposure was performed in the same way as for the SSH-metals in regard to concentrations (EC_50_ and EC_90_ [7]) and duration (2, 4 and 8 days). Similarly, two different RNA pools were obtained: one from organisms exposed to the pesticides and one from control organisms. The exposure concentrations of all compounds are given in table 1.

**Table 1:** Concentrations of the four metals and four pesticides to which *E. albidus* were exposed for the SSH libraries development.

	EC_50_ (mg/kg)	EC_90_ (mg/kg)	References
*SSH Metals*			
Copper	100	320	[5]
Cadmium	6	150	[27]
Zinc	40	100	[27]
Nickel	50	120	[28]
*SSH Pesticides*			
Dimethoate	2	25	[7]
Atrazine	3	50	[7]
Carbendazim	0.5	3	[7]
Lindane	40	130	[7]

EC_50_ = Concentration that induces 50% reduction in the number of juveniles; EC_90_ = Concentration that induces 90% reduction in the number of juveniles.

Concentrations of exposure are based on the effect concentrations on reproduction, available on the literature.

To each RNA pool, 0.1 volumes of 3 M sodium acetate and 3 volumes of 96% ethanol were added and the pairs of pools were shipped at room temperature to Evrogen (Moscow, Russia). Amplification of the double stranded cDNAs (using SMART approach [29]) and the subtraction procedures were performed by Evrogen for both libraries. The cDNA was SMART-amplified (19 cycles), starting from 0.5 µg of each RNA pool, and used for subtractive hybridization using SSH method in both directions [24], [25]. Prior to the library construction, the samples were subjected to the mirror orientation selection (MOS) procedure [30] to eliminate false positive clones resulting from the SSH procedures (Evrogen). The treated samples were then handled by us for the libraries construction. Briefly, the subtracted cDNAs were ligated in a TA-vector system (pGEM-T easy vector, Promega). *Escherichia coli* calcium competent cells (JM109, Promega) were transformed through heat shock. The recombinant clones were picked and grown in 96-well plates. Glycerol stocks were made (12.5%) and stored at −80°C. Clones were amplified with vector-specific primers (T7 and SP6 primers, Promega), and purified by an exosap reaction [31] based on exonuclease I and shrimp alkaline phosphatase (Fermentas).

### EST Sequencing and Comparative Sequence Analysis

From the SSH libraries, 1920 clones were selected (960 from each library). After checking the quality of the PCR inserts on an agarose gel, 67 clones had no inserts or had more than one insert and were excluded. Therefore, the remaining 1853 purified clones were sent to be sequenced with primers SP6 and T7 (VIB service, Flemish Institute for Biotechnology).

CodonCode Aligner software (www.codoncode.com/aligner) was used to remove vectors and screen for low-quality sequence regions. From the 1853, 101 sequences were shorter than 50 base pairs (bp) or did not pass the quality control (low-quality sequence regions only) and were thus removed from further analysis. In sum, from the 921 clones sequenced from the metals enriched library, we obtained 875 good quality sequences (95%) and from the 932 clones sequenced from the pesticides enriched library, we obtained 877 good quality sequences (94%). All good quality sequences were submitted to GenBank dbEST (accession numbers: JK309883-JK310757; JK474167 - JK475043).

**Figure 2:**
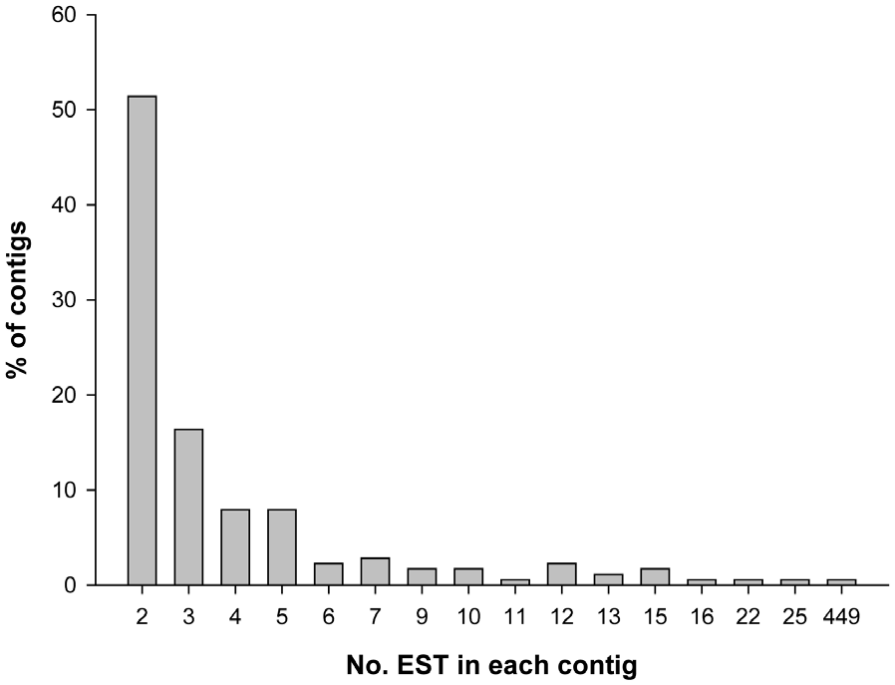
Expressed Sequence Tags distribution over the 177 contigs.

Sequences from the first cDNA library developed by Amorim and co-authors [16] were added for further analysis. In total 2100 ESTs were retrieved from the three libraries and aligned and assembled using Cap3 program (http://www.genome.clemson.edu/cgi-bin/cugi_cap3). This procedure resulted in 1124 unique sequences (clusters): 947 singletons (338 ESTs from the normalized library, 370 ESTs from the metals enriched library and 239 EST from the pesticides enriched library) and 177 contigs. The 45% singletons obtained in this study is inferior to the 80% observed in the EST sequencing project for *Eisenia fetida* [19] but similar to the percentages of singletons observed in the EST sequencing projects of other terrestrial invertebrate species: 49% for *Eisenia andrei* [32], 52% for *Folsomia candida* [20] or 53% for *Lumbricus rubelus* [18]. From the 177 contigs, nearly 85% were assembled from 2 to 5 sequences and more than half were assembled from only 2 sequences (Figure 2).

The length of the 177 contigs varied from 69 to 1630 bp with an average of 735 bp. The highest number of sequences in one contig was by far observed on the pesticides enriched library with 449 ESTs, whereas the highest depth among the contigs in the metals enriched library was 24 ESTs and in the normalized library was 4 ESTs. In terms of redundancy [total number of sequences divided by the number of clusters [20]], the pesticides library was the most redundant (3.22) followed by the metals library (1.80) and the least one, the normalized library (1.02). Overall, data had a redundancy of 1.87. Interestingly, also Timmermans et al. [20] refer a similar difference in redundancy obtained for the phenantrene (3.18) library in comparison to the cadmium (1.62) and normalized (1.32) ones.

**Figure 3:**
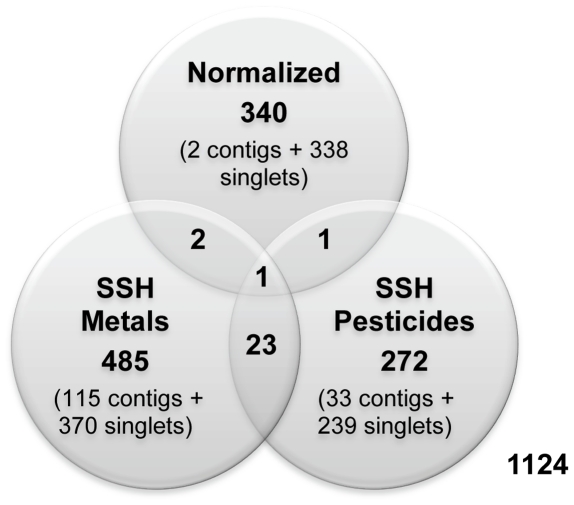
Cluster overlaps between the three different libraries. The common clusters represent contigs assembled from ESTs with different library origins. Normalized: normalized library; SSH Metals: Library enriched for genes differentially expressed after metal exposures; SSH Pesticides: Library enriched for genes differentially expressed after pesticide exposures.

The overlaping ESTs from the different cDNA libraries is represented in Figure 3. Interestingly, very little overlap occurred, with only one contig containing sequences from the three libraries (cluster EAC00169, Table 2). This was also observed in other studies (e.g. [20]) confirming the relevance of the enrichment with as much varied conditions as possible.

**Table 2:** The most represented sequenced transcripts in *E. albidus* cDNA libraries.

Cluster ID	ESTs	Length	Library	Blast Hit	E-value	GeneBank AccNumber
EAC00048	449	660	Pest	No significant hit	–	–
EAC00024	25	480	Met+Pest	Actin	7.25E-12	ADJ56346
EAC00129	22	960	Met+Pest	Sarcoplasmic calcium-binding protein	1.01E-09	P04572
EAC00065	16	720	Pest	No significant hit	–	–
EAC00139	15	1620	Met	Actin	1.04E-17	NP_001003349
EAC00074	15	660	Pest	myosin heavy chain	5.51E-07	CAC28360
EAC00083	15	840	Pest	No significant hit	–	–
EAC00094	13	1260	Met	No significant hit	–	–
EAC00138	13	1140	Met	No significant hit	–	–
EAC00108	12	720	Met+Pest	myosin heavy chain	3.11E-06	AAD52842
EAC00109	12	840	Met	No significant hit	–	–
EAC00157	12	720	Met	ADP/ATP carrier protein 3	1.34E-11	NP_001187478
EAC00066	12	720	Pest	No significant hit	–	–
EAC00070	11	540	Pest	MADS FLC-like protein 2	4.33E-05	ACL54966
EAC00035	10	660	Met+Pest	hemoglobin c chain precursor	3.30E-06	CAA09958
EAC00092	10	780	Met	No significant hit	–	–
EAC00143	10	660	Met+Pest	ribosomal protein s7	8.78E-14	AAW50967
EAC00169	9	1020	Met+Pest+Norm	Myosin regulatory light chain	4.12E-12	P80164
EAC00015	9	900	Met	No significant hit	–	–
EAC00082	9	720	Pest	myosin heavy chain cg17927	3.44E-05	EFA08290

Met: sequences from the SSH library enriched for genes differentially expressed after metal exposures; Pest: sequences from the SSH library enriched for genes differentially expressed after pesticide exposures; Norm: sequences from the normalized library.

The sequenced unique fragments (singletons and consensus sequences of assembled contigs) were identified based on their similarity to sequences in the National Centre for Biotechnology Information (NCBI) database as determined by the Basic Local Alignment Search Tool (BLAST) [33]. The sequences were submitted to Blast2GO [34] being compared with peptide sequence databases using BLASTX analysis. From the 1124 clusters, a total of 459 sequences (41%) matched known proteins in the database with an e-value ≤ 10^−5^. Among these, 72 sequences (16%) had e-values between 10^−123^ and 10^−50^.

The most abundant sequenced transcripts identified were actin, myosin, Sarcoplasmic calcium-binding protein, ADP/ATP carrier protein 3, MADS FLC-like protein 2, hemoglobin c and ribosomal protein s7 (Table 2).

As can be seen in table 2, housekeeping genes like actin were highly represented in the SSH libraries but not in the normalized library, indicating that the normalization method was efficient [20], [35].

From the 459 blast hits, 46 (10%) matched sequences from earthworms, soil organisms phylogenetically close to *E. albidus* (*Eisenia fetida*, *Lumbricus rubellus*, *Lumbricus terrestris*, *Lumbricus variegatus*).

### Functional Annotation

Gene ontology terms (GO) were assigned to the predicted proteins by homology blast using the same Blast2GO software [34]. A total of 415 sequences had at least one GO term assigned (37% of the 1124 clusters).

The summary of GO terms showing the representation of the higher-level terms (GO-slim), assigned to 5 or more sequences is given in table 3.

**Table 3:** GO-slim terms represented by more than 5 sequences in the combined library datasets for *E. albidus*.

	Gene Ontology ID	No. Sequences	Library(no. sequences)
**Biological process**			
Lipid metabolic process	GO:0006629	5	Met(3)+Norm(2)
Cellular amino acid and derivative metabolic process	GO:0006519	5	Met(4)+Pest(1)
Reproduction	GO:0000003	6	Pest(2)+Norm(4)
Carbohydrate metabolic process	GO:0005975	7	Met(4)+Pest(3)
Cell proliferation	GO:0008283	8	Met(5)+Norm(3)
Ion Transport	GO:0006811	8	Met(8)
Cell death	GO:0008219	8	Met(6)+Pest(2)
Signal transduction	GO:0007165	9	Met(3)+Pest(2)+Norm(4)
Protein modification process	GO:0006464	11	Met(4)+Pest(3)+Norm(4)
Growth	GO:0040007	12	Met(5)+Pest(3)+Norm(4)
Cell cycle	GO:0007049	12	Met(6)+Pest(4)+Norm(2)
Response to stress	GO:0006950	13	Met(8)+Pest(1)+Norm(4)
Embryonic development	GO:0009790	14	Met(7)+Pest(3)+Norm(4)
Catabolic process	GO:0009056	18	Met(11)+Pest(5)+Norm(2)
Cytoskeleton organization	GO:0007010	18	Met(14)+Pest(4)
Cell differentiation	GO:0030154	22	Met(17)+Pest(3)+Norm(2)
Generation of precursor metabolites and energy	GO:0006091	23	Met(13)+Pest(6)+Norm(4)
Anatomical structure morphogenesis	GO:0009653	28	Met(21)+Pest(4)+Norm(3)
Translation	GO:0006412	35	Met(20)+Pest(7)+Norm(8)
Transcription	GO:0006350	72	Met(5)+Pest(65)+Norm(2)
**Molecular function**			
Kinase activity	GO:0016301	5	Met(2)+Pest(2)+Norm(1)
Translation factor activity, nucleic acid binding	GO:0008135	8	Met(3)+Pest(3)+Norm(2)
Electron carrier activity	GO:0009055	8	Met(4)+Pest(1)+Norm(3)
Transcription regulator activity	GO:0030528	8	Met(5)+Pest(2)+Norm(1)
Actin binding	GO:0003779	12	Met(4)+Pest(8)
Peptidase activity	GO:0008233	13	Met(7)+Pest(4)+Norm(2)
Transporter activity	GO:0005215	16	Met(12)+Pest(1)+Norm(3)
Calcium ion binding	GO:0005509	17	Met(9)+Pest(6)+Norm(2)
Motor activity	GO:0003774	18	Met(4)+Pest(13)+Norm(1)
RNA binding	GO:0003723	21	Met(10)+Pest(3)+Norm(8)
Structural molecule activity	GO:0005198	45	Met(31)+Pest(5)+Norm(9)
DNA binding	GO:0003677	68	Met(3)+Pest(64)+Norm(1)
Nucleotide binding	GO:0000166	82	Met(51)+Pest(28)+Norm(3)
**Cellular Component**			
Nucleolus	GO:0005730	7	Met(4)+Norm(3)
Nucleoplasm	GO:0005654	8	Met(4)+Pest(2)+Norm(2)
Extracellular region	GO:0005576	8	Met(7)+Norm(1)
Plasma membrane	GO:0005886	10	Met(6)+Norm(4)
Mitochondrion	GO:0005739	26	Met(15)+Pest(4)+Norm(7)
Ribosome	GO:0005840	29	Met(18)+Pest(3)+Norm(8)
Cytosol	GO:0005829	34	Met(21)+Pest(6)+Norm(7)
Protein complex	GO:0043234	64	Met(35)+Pest(22)+Norm(7)
Cytoskeleton	GO:0005856	82	Met(50)+Pest(29)+Norm(3)

Met: sequences from the SSH library enriched for genes differentially expressed after metal exposures; Pest: sequences from the SSH library enriched for genes differentially expressed after pesticide exposures; Norm: sequences from the normalized library.

Transcription and translation are the most represented biological processes in the developed libraries. As for the molecular functions and cellular components, the most represented are the nucleotide and DNA binding and the cytoskeleton and protein complex, respectively. Most of the biological functions have a higher representation in the metals enriched library, with the exception of reproduction which is not represented in this library and transcription which is mainly represented in the pesticides enriched library.

Molecular pathways were assigned to the clusters using the Kyoto Encyclopedia of Genes and Genomes (KEGG) [36] based on their Enzyme Commission numbers (EC). In total, 61 sequences matched enzymes with an EC number (5.5% of the 1124 clusters), belonging to 25 different pathways, all related to metabolism. The metabolisms with more pathways where the enzymes coded for *E. albidus* sequences are involved were the energy and amino acid metabolisms, followed by e.g. the metabolisms of carbohydrates and nucleotides (Table 4).

**Table 4:** KEGG pathways in the combined library datasets for *E. albidus*.

	No. Sequences	Library (no. sequences)
**Amino acid metabolism**	**12**	
Alanine, aspartate and glutamate metabolism	1	Met(1)
Arginine and proline metabolism	2	Met(1)+Pest(1)
Cysteine and methionine metabolism	2	Met(2)
Glycine, serine and threonine metabolism	3	Met(2)+Pest(1)
Phenylalanine metabolism	1	Met(1)
Phenylalanine, tyrosine and tryptophan biosynthesis	1	Met(1)
Tyrosine metabolism	2	Met(2)
**Energy metabolism**	**25**	
Carbon fixation in photosynthetic organisms	3	Met(2)+Pest(1)
Methane metabolism	4	Met(4)
Oxidative phosphorylation	14	Met(9)+Pest(1)+Norm(4)
Photosynthesis	4	Met(4)
**Carbohydrate metabolism**	**3**	
Glycolysis/Gluconeogenesis	2	Pest(2)
Pentose phosphate pathway	1	Met(1)
**Nucleotide metabolism**	**3**	
Purine metabolism	2	Met(2)
Pyrimidine metabolism	1	Met(1)
**Lipid metabolism**	**1**	
Glycerophospholipid metabolism	1	Met(1)
**Glycan Biosynthesis and Metabolism**	**2**	
N-Glycan biosynthesis	1	Met(1)
Various types of N-glycan biosynthesis	1	Met(1)
**Metabolism of Cofactors and Vitamins**	**2**	
Nicotinate and nicotinamide metabolism	1	Met(1)
Thiamine metabolism	1	Met(1)
**Biosynthesis of Other Secondary Metabolites**	**3**	
Isoquinoline alkaloid biosynthesis	1	Met(1)
Novobiocin biosynthesis	1	Met(1)
Tropane, piperidine and pyridine alkaloid biosynthesis	1	Met(1)
**Metabolism of Terpenoids and Polyketides**	**1**	
Biosynthesis of ansamycins	1	Met(1)
**Xenobiotics Biodegradation and Metabolism**	**1**	
Styrene degradation	1	Met(1)

Met: sequences from the SSH library enriched for genes differentially expressed after metal exposures; Pest: sequences from the SSH library enriched for genes differentially expressed after pesticide exposures; Norm: sequences from the normalized library.

### Development of the Database

EnchyBASE development required the integration of diverse bioinformatics software. Four intertwined components were needed to deploy the whole system: a web application server, a database management system and a local BLAST tool.

The ESTs and associated annotation information led to deployment of EnchyBASE in an Apache Web Server with PostgreSQL for the database backend. PartiGene [37], the chosen gene sequence-clustering tool, is the key responsible for the adopted solutions. Its web component, wwwPartiGene, requires serving dynamic PHP pages and a connection to a PostgreSQL database. ViroBLAST [38] was selected as a local BLAST tool as it provides an eased setup process for executing various distinct BLASTs against local sequence clusters.

Whereas the miscellaneous system components were relatively easy to adapt or implement, the constructed integration pipeline was a more complex task. EnchyBASE deployment workflow involved three key steps: 1) sequence annotation, 2) sequence clustering and annotation of clusters, and 3) BLAST database migration.

The first step involved the annotation of the obtained ESTs for *E. albidus* using the BLAST2GO bioinformatics tool [34]. Sequences were clustered using Cap3 program and the retrieved clusters were also annotated. Generated data was then moved on to PartiGene. At last, annotated sequences were used to generate a BLASTable database using NCBI BLAST toolkit [39]. The resulting dataset was made available to ViroBLAST for real time BLAST against *E. albidus* sequence data.

For end-users, the system provides three main key features: sequence download, annotation search and BLAST. Researchers are able to download the entire sequence dataset or specific clustered sequences. The search engine allows browsing data through multiple queries. Users can search for specific clusters or sequences, common BLAST annotations, ontology annotations and primer features. At last, BLASTing can be performed against *E. albidus* data. Available BLAST functions are blastn, blastx, tblastn and tblastx. BLAST parameters may be easily configured in EnchyBASE’s BLAST interface.

## Discussion

Various advantages may be pointed in regard to the genomic information gathered in the course of this study. With the development of EnchyBASE users can access the sequences present in each of the libraries, as well as all the information related to each sequence (BLAST homologies and GO terms) when available. This information can be downloaded after simple search queries by Cluster ID, GenBank accession number, BLAST annotation or GO term. Furthermore, designed primers with tested efficiencies for some of the sequences are provided. The database also enables the users to run blasts with their own sequences and look for homologies with the enchytraeids species.

In the present work it was possible to observe that only one gene was shared by the three libraries and, in general the gene overlap between libraries was low (Figure 3). Also, the exposure to the two different groups of chemicals (metals and pesticides) affected distinct biological functions e.g. reproduction or lipid metabolic processes were only affected by pesticides or metals, respectively (Table 3).

These findings suggest that the exposure to pesticides triggered a different set of genes in comparison to metals exposure. However, the actual expression profiles of *E. albidus*, when exposed to the individual chemicals or natural stressors, require confirmation through experiments of gene expression analysis.

A new custom Agilent microarray was developed, with printed 60-mer oligonucleotides designed from the unique sequences in the database. All transcription data generated with this microarray will be stored in enchyBASE similarly to what is presently done with the data gathered with the former cDNA microarray, where information on the differentially expressed genes to each stress condition is available. This information, along with the respective differentially expressed gene sequences, can be used by the scientific community in functional genomics studies and quantitative polymerase chain reaction (qPCR) experiments.

In the near future and through hybridizations on this microarray we expect to increase knowledge on the molecular pathways involved in response to stress factors. This information can improve the current understanding of chemicals mode of action on soil invertebrates, which along with data on other organisms can help to develop predictive models of toxic effects. Additionally, generating specific stress signature fingerprints would be of particular interest to classify different types of stressors, levels of toxicity, or chemical groups.

The obtained sequence information can be potentially used to answer questions regarding chemical exposure as e.g. adaptation to chemical stress. It is commonly agreed that soil invertebrates can genetically adapt to metal stress, modifying metal toxicity and gaining resistance to contaminated soils [40]–[42]. Some known mechanisms of detoxification like the storage of metal ions in membrane enclosed cellular granules or in metallothionein complexes [40]–[44] have been associated with changes in the energy metabolism due to the need of energy for these detoxification processes [40]. The use of transcriptomics to determine differential gene expression in metal tolerant populations has recently been successfully applied in the soil arthropod *Orchesella cincta* [45], [46]. Roelofs and co-authors observed different gene expression patterns between reference and tolerant populations of this species after cadmium exposure, confirming the micro-evolutionary processes occurring in this soil species’ populations [46]. Mechanisms of genetic adaptation to metal stress in enchytraeids has never been observed but can now be investigated with similar transcription studies using enchyBASE and the newly developed microarray.

These new tools can also be potentially used to answer other ecological questions, e.g. drought tolerance. Maraldo and co-authors [47], [48] found that *E. albidus* is able to adapt to environments with strong fluctuations in humidity, being able to keep its water content stable during moist and relatively dry conditions. It is known that *E. albidus* can even tolerate incredibly low temperature (−20°C), probably related to the ability to synthesise high concentrations of glucose [49]. Mechanisms of tolerance to drought, freeze and other environmental conditions, have been currently studied for the springtails *Onychiurus arcticus* [50] and *Folsomia candida* [51]–[53]. Such can also be further studied in enchytraeids using transcriptomic tools.

Also, *E. albidus* is known to be able to avoid unfavourable conditions such as natural stressors like inadequate soil properties (e.g. pH, clay content) [12] or chemicals [8], [54]. Interestingly, not all chemicals are equally avoided, and some are even not perceived despite their high toxicity. The underlying mechanisms of these differences can also be pursued with transcription studies. Among other potential utility of EnchyBASE is the study of the mechanisms behind chemical mixtures toxicity or combinations of environmental stressors, relevant issues in soil ecotoxicology.

The microarray and EnchyBASE provide the scientific community information with potentially multiple applications, constituting a stepping stone for ecotoxicology, genomics and molecular ecological studies with enchytraeids.
